# Plasma amyloid-β ratios in autosomal dominant Alzheimer’s disease: the influence of genotype

**DOI:** 10.1093/brain/awab166

**Published:** 2021-04-23

**Authors:** Antoinette O'Connor, Josef Pannee, Teresa Poole, Charles Arber, Erik Portelius, Imogen J Swift, Amanda J Heslegrave, Emily Abel, Nanet Willumsen, Helen Rice, Philip S J Weston, Natalie S Ryan, James M Polke, Jennifer M Nicholas, Simon Mead, Selina Wray, Lucía Chávez-Gutiérrez, Chris Frost, Kaj Blennow, Henrik Zetterberg, Nick C Fox

**Affiliations:** 1 Dementia Research Centre, UCL Queen Square Institute of Neurology, London, WC1N 3BG, UK; 2 UK Dementia Research Institute at UCL, London, WC1E 6AU, UK; 3 Department of Psychiatry and Neurochemistry, Institute of Neuroscience and Physiology, Sahlgrenska Academy at University of Gothenburg, S-431 80 Mölndal, Sweden; 4 Clinical Neurochemistry Laboratory, Sahlgrenska University Hospital, S-431 80 Mölndal, Sweden; 5 Department of Medical Statistics, London School of Hygiene and Tropical Medicine, London, WC1E 7HT, UK; 6 Department of Neurodegenerative Disease, UCL Queen Square Institute of Neurology, London, WC1N 3BG, UK; 7 Neurogenetics Laboratory, National Hospital for Neurology and Neurosurgery, University College London Hospitals NHS Foundation Trust, London WC1N 3BG, UK; 8 National Prion Clinic, National Hospital for Neurology and Neurosurgery, University College London Hospitals NHS Foundation Trust, London WC1N 3BG, UK; 9 MRC Prion Unit at UCL, UCL Institute of Prion Diseases, 33 Cleveland Street, London W1W 7FF, UK; 10 VIB-KU Leuven Center for Brain and Disease Research, 3000 Leuven, Belgium; 11 Department of Neurosciences, Leuven Research Institute for Neuroscience and Disease (LIND), KU Leuven, 3000 Leuven, Belgium

**Keywords:** autosomal dominant Alzheimer’s disease, blood biomarkers, dementia, amyloid-beta

## Abstract

*In vitro* studies of autosomal dominant Alzheimer’s disease implicate longer amyloid-β peptides in disease pathogenesis; however, less is known about the behaviour of these mutations *in vivo*. In this cross-sectional cohort study, we used liquid chromatography-tandem mass spectrometry to analyse 66 plasma samples from individuals who were at risk of inheriting a mutation or were symptomatic. We tested for differences in amyloid-β (Aβ)42:38, Aβ42:40 and Aβ38:40 ratios between presenilin 1 (*PSEN1*) and amyloid precursor protein (*APP*) carriers. We examined the relationship between plasma and *in vitro* models of amyloid-β processing and tested for associations with parental age at onset. Thirty-nine participants were mutation carriers (28 *PSEN1* and 11 *APP*)*.* Age- and sex-adjusted models showed marked differences in plasma amyloid-β between genotypes: higher Aβ42:38 in *PSEN1* versus *APP* (*P* < 0.001) and non-carriers (*P* < 0.001); higher Aβ38:40 in *APP* versus *PSEN1* (*P* < 0.001) and non-carriers (*P* < 0.001); while Aβ42:40 was higher in both mutation groups compared to non-carriers (both *P* < 0.001). Amyloid-β profiles were reasonably consistent in plasma and cell lines. Within the *PSEN1* group, models demonstrated associations between Aβ42:38, Aβ42:40 and Aβ38:40 ratios and parental age at onset. *In vivo* differences in amyloid-β processing between *PSEN1* and *APP* carriers provide insights into disease pathophysiology, which can inform therapy development.

## Teaser

O’Connor *et al.* reveal significant differences in plasma Aβ ratios between *PSEN1 and APP* carriers, broadening understanding of the molecular drivers of Alzheimer’s disease. They also show associations between higher Aβ42:40 and Aβ42:38 ratios and earlier disease onset, supporting the pathogenicity of these peptide ratios.

## Introduction

Understanding Alzheimer’s disease pathogenesis is critical to realizing disease-modifying treatments. Autosomal dominant Alzheimer’s disease (ADAD), caused by mutations in presenilin 1/2 (*PSEN1*/*2*) or amyloid precursor protein (*APP*), is a valuable model for characterizing the molecular drivers of Alzheimer’s disease.^1^

PSEN1, the catalytic subunit of γ-secretase, sequentially cuts APP: initial endopeptidase cleavage generates an amyloid-β (Aβ) peptide, either Aβ49 (major product) or Aβ48 (minor product).^2^ Subsequent proteolysis largely occurs down two pathways: Aβ49 > 46 > 43 > 40 or Aβ48 > 45 > 42 > 38.^3^ As Aβ49 is the predominant endopeptidase cleavage product, normal APP processing largely leads to Aβ40 formation.^2^ Pathogenic ADAD mutations alter APP processing resulting in more and/or longer, aggregation prone, amyloid-β peptides, which accelerate cerebral amyloid accumulation leading to typical symptom onset in a person’s thirties to fifties.^4^^,^^5^

Both *APP* and *PSEN1*/*2* mutations increase production of longer (e.g. Aβ42) relative to shorter (e.g. Aβ40) peptides.^5^ However, there are intriguing inter-mutation differences in amyloid-β profiles. *PSEN1* mutant lines produce increased Aβ42:38 ratios reflecting impaired γ-secretase processivity.^5^^,^^6^ In contrast, *APP* mutations at the γ-secretase cleavage site increase Aβ38:40 ratios, consistent with preferential processing down the Aβ48 pathway.^6^ So far, studies examining the influence of ADAD genotypes on amyloid-β ratios *in* *vivo* have been lacking.

Increasingly sensitive mass spectrometry-based assays now make it possible to measure concentrations of different amyloid-β moieties in plasma.^7^ Therefore, we aimed to analyse plasma amyloid-β levels in an ADAD cohort, explore influences of genotype and clinical stage, and examine relationships between ratios and both parental age at onset (AAO) and estimated years to/from symptom onset (EYO), while also assessing consistency with *in* *vitro* models of amyloid-β processing.

## Materials and methods

### Study design and participants

We recruited 66 participants from the longitudinal ADAD study at University College London (UCL); details have been described previously.^1^ Samples were collected from August 2012 to July 2019 and concomitantly a semi-structured health questionnaire and Clinical Dementia Rating (CDR) scale were completed.^8^ EYO was calculated by subtracting parental AAO from the participant’s age. Participants were defined as symptomatic if global CDR was >0. ADAD mutation status, determined using Sanger sequencing, was provided only to statisticians, ensuring blinding of participants and clinicians. The study had local Research Ethics Committee approval and written informed consent was obtained from all participants or a consultee.

### Measurement of plasma amyloid-β levels

EDTA plasma samples were processed, aliquoted and frozen at −80°C according to standardized procedures and shipped frozen to the Clinical Neurochemistry Laboratory, Sahlgrenska University Hospital, for analysis blinded to participants’ mutation status and diagnosis. Samples were analysed using a liquid chromatography-tandem mass spectrometry method using an optimized protocol for immunoprecipitation for improved analytical sensitivity (Supplementary Figs 1 and 2).^9^ Pooled plasma samples were used to track assay performance; intra- and inter-assay coefficients of variation were <5%.

### Correlation of amyloid-β ratios in plasma and in induced pluripotent stem cell neurons

A sub-study investigated the consistency of amyloid-β profiles between plasma and induced pluripotent stem cell (iPSC)-derived neurons. Amyloid-β profiles were compared based on mutation for eight iPSC lines; data from six iPSC lines ave been previously reported by Arber *et al.*^6^ Mutations tested were *APP* V717I (*n* = 2), *PSEN1* intron 4 (*n *= 1), Y115H (*n* = 1), M139V (*n* = 1), R278I (*n* = 1) and E280G (*n* = 2). Plasma and iPSC samples were from the same participant or, where matched plasma was unavailable, plasma from a carrier of the same mutation and, if possible, a family member. Aβ42:40, Aβ38:40 and Aβ42:38 ratios were normalized by taking the ratio of the value for each mutation carrier to the control median for each experimental setting (*n* = 27 non-carriers for plasma, *n* = 5 iPSC controls lines from non-ADAD families) (Supplementary Table 1, ratio values).

IPSC-neuronal amyloid-β was quantified as previously reported Arber *et al.*^6^ Briefly, iPSCs were differentiated to cortical neurons for 100 days and then 48 h-conditioned culture supernatant was centrifuged removing cell debris. Amyloid-β was analysed via electrochemiluminescence on the MSD V-Plex Aβ peptide panel (6E10), according to the manufacturer’s instructions.

### Statistical analysis

**Figure 1:**
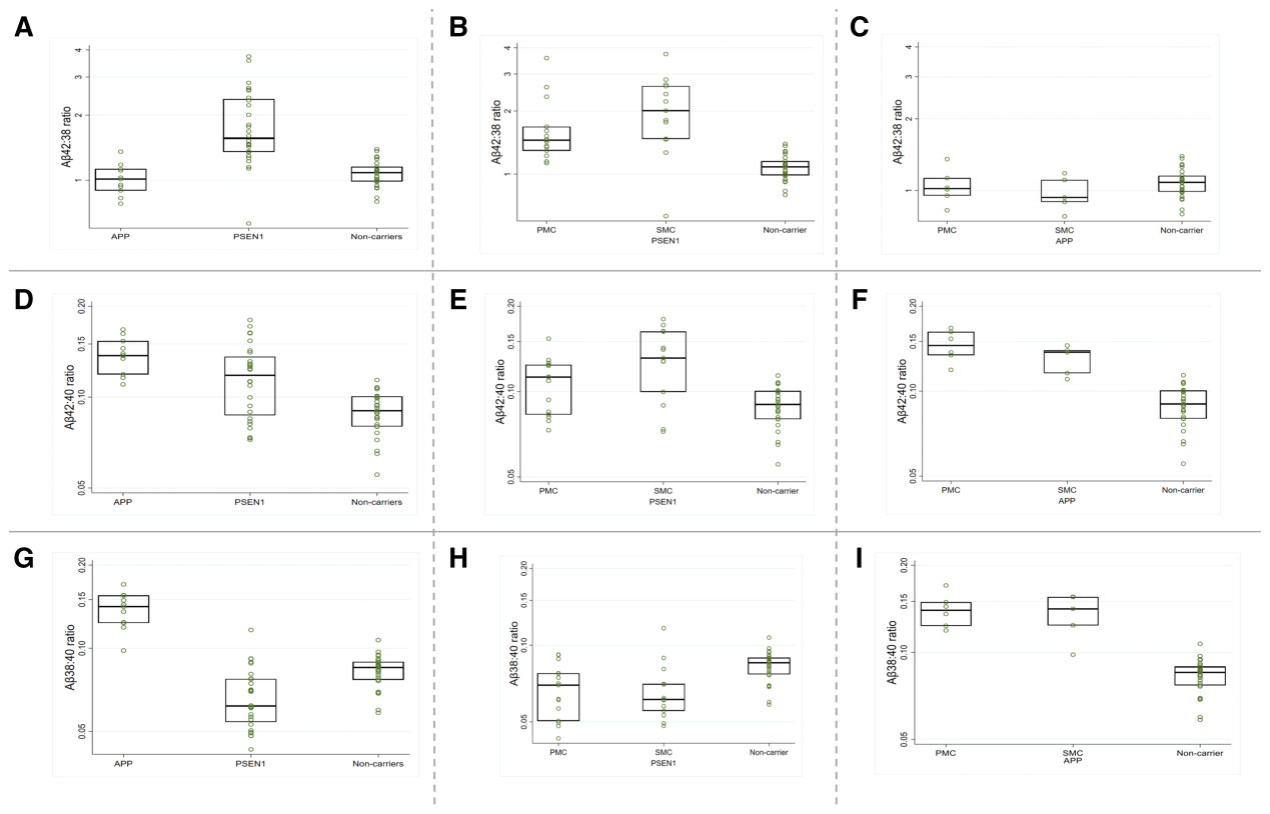
**Box plots for observed plasma amyloid-β ratios.** (**A**–**C**) Plasma Aβ42:38, (**D**–**F**) Aβ42:40 and (**G**–**I**) Aβ38:40 ratios are shown with the *y*-axis on a logarithmic scale. Mutation carriers were divided into (**A**,** D** and** G**) *APP* and *PSEN1* carriers and non-carriers; (**B**, **E **and** H**) *PSEN1* presymptomatic and symptomatic mutation carriers and non-carriers and (**C**,** F **and **I**) *APP* presymptomatic and symptomatic mutation carriers and non-carriers. Boxes show the median and first and third quartiles. Dots represent individual observations.

Summary descriptive statistics were calculated by mutation type (*PSEN1*, *APP*, non-carriers) and box plots produced for Aβ42:38, Aβ38:40 and Aβ42:40 ratios. Box plots were presented by mutation type (*PSEN1* versus *APP* versus non-carriers), and then individually for *PSEN1* and *APP* carriers by clinical stage (presymptomatic versus symptomatic versus non-carriers) (Fig. 1). Amyloid-β ratios are displayed on logarithmic scales. Age- and sex-adjusted differences were estimated between mutation type for each ratio, as were differences by clinical stage for each ratio, separately for *APP* and *PSEN1* carriers. These comparisons were made using mixed models including random intercepts for clusters comprising individuals from the same family and group, with random intercept and residual variances allowed to differ for the groups being compared. Pairwise comparisons were only carried out if a joint test provided evidence of differences. Ratios were log-transformed; estimated coefficients were back-transformed to multiplicative effects.

The relationship between parental AAO, EYO and age (EYO = age − AAO) means that it is not possible to estimate separate effects of AAO and EYO on amyloid-β ratios adjusting for age using a conventional statistical analysis: if age is held constant, then a 1-year increase in AAO implies a 1-year decrease in EYO and vice versa, hence their effects are aliased. However, the aim here should be to allow for ‘normal ageing’ (as observed in non-carriers), and this is possible. For each combination of mutation carrier group (*PSEN1* and *APP*) and amyloid-β ratio a separate mixed model was fitted jointly to the carrier group and the non-carrier group. Each model allowed the logarithm of the amyloid-β ratio to depend on AAO, EYO and sex (but not age) in the carrier group, and on just sex and age (estimating ‘normal ageing’) in the non-carrier group. Random effects were included as in the between group comparisons above. In the carrier group, the effect of AAO adjusted for EYO, sex and (non-carrier) ‘normal ageing’ was obtained by subtracting the ‘normal ageing’ effect from the AAO effect (adjusted for sex and EYO). Analogously the effect of EYO adjusted for AAO, sex and ‘normal ageing’ was obtained by subtracting the ‘normal ageing’ effect from the EYO effect (adjusted for sex and AAO) in the carrier group. For Aβ42:38 in *PSEN1* carriers there was evidence also to include a quadratic term for parental AAO. For each analysis, the estimated geometric mean ratio (and 95% confidence interval, CI) was plotted against parental AAO, standardizing to an equal mix of males/females, an EYO of 0 (i.e. the point of symptom onset), and adjusted for ‘normal ageing’ relative to age 43 (the average age of mutation carriers). Analogous plots of estimated geometric mean ratio (and 95% CI) against EYO were standardized to an equal mix of males/females, an AAO of 43 (average age of mutation carriers) and adjusted for ‘normal ageing’ relative to age 43.

Spearman correlation coefficients were calculated to assess the association between plasma and iPSC-neuron amyloid-β ratios.

Analyses were performed using Stata v.16.

### Data availability

Data are available on reasonable request from qualified investigators, adhering to ethical guidelines.

## Results

Demographic and clinical characteristics are presented in Table 1 for the 27 non-carriers and 39 mutation carriers (28 *PSEN1*, 11 *APP*). Mutation details are provided in Supplementary Table 2.

**Table 1:** Baseline characteristics (*n* = 66)

	**Non-carrier** ***n* = 27**	***APP*** ***n* = 11**	***PSEN1*** ***n* = 28**
Sex^a^, *n* (%)			
Female	16 (59)	3 (27)	15 (54)
Male	11 (41)	8 (73)	13 (46)
Age^b^, years, mean (SD)	39.6 (10.4)	46.5 (12.5)	43.0 (8.7)
Stage *n* (%)^c^	N/A	–	–
Presymptomatic	–	6 (54.6)	15 (53.6)
Symptomatic	–	5 (45.4)	13 (46.4)
Aβ1–42^d^, pg/ml, median (IQR)	20.3 (18.3–24.5)	29.5 (24.2–36.0)	26.3 (14.7–32.3)
Aβ1–40^e^, pg/ml, median (IQR)	225.7 (212.2–246.1)	214.0 (174.5–232.8)	221.5 (146.5–252.2)
Aβ1–38^f^, pg/ml, median (IQR)	19.2 (16.7–21.0)	27.0 (24.8–35.6)	14.1 (9.6–18.4)
Aβ1–42/1–40 ratio, median (IQR)	0.09 (0.08–0.10)	0.14 (0.12–0.15)	0.12 (0.09, 0.14)
Aβ1–42/1–38 ratio, median (IQR)	1.08 (0.99–1.15)	1.01 (0.90–1.13)	1.56 (1.36–2.37)
Aβ 1–38/1–40 ratio, median (IQR)	0.09 (0.08–0.09)	0.14 (0.12–0.16)	0.06 (0.05–0.08)

^a^ No evidence of a difference between groups: Fisher’s exact test *P* = 0.21.

^b^ No evidence of a difference between groups: Wald test *P* = 0.14.

^c^ All non-carriers were asymptomatic.

^d^ For Aβ 1–42 there was evidence of a difference between groups (Wald test *P* = 0.0003), after adjusting for age and sex. Mean Aβ 1–42 in *APP* carriers was an estimated adjusted 10.4 pg/ml higher (95% CI 5.1, 15.7, *P* < 0.001) than non-carriers and in *PSEN1* was 5.3 pg/ml higher (95% CI: 0.5, 10.1, *P* = 0.03) than non-carriers, while there was no evidence of a difference between *APP* carriers and *PSEN1* carriers (*P* = 0.10).

^e^ For Aβ 1–40 there was no evidence of a difference between groups after adjusting for age and sex: Wald test *P* = 0.61.

^f^ For Aβ 1–38 there was evidence of a difference between groups (Wald test *P* < 0.0001) after adjusting for age and sex. Mean Aβ 1–38 in *APP* carriers was an estimated adjusted 14.9 pg/ml higher (95% CI 8.7, 21.1; *P* < 0.001) than PSEN1 carriers and 10.2 pg/ml higher (95% CI: 4.1, 16.3; *P* = 0.001) than non-carriers, and in PSEN1 carriers was 4.7 pg/ml lower (95% CI: 2.0, 7.4; *P* = 0.001) than non-carriers.

Age- and sex-adjusted models showed marked differences in plasma amyloid-β between *PSEN1* and *APP* carriers. The geometric mean of Aβ42:38 was higher in *PSEN1* compared to both *APP* carriers (69% higher, 95% CI: 39%, 106%; *P *<* *0.001) and non-carriers (64% higher, 95% CI: 36%, 98%; *P *<* *0.001), while there was no evidence of a difference between *APP* carriers and non-carriers (*P *=* *0.60) (Fig. 1A).

Plasma Aβ42:40 was raised in both *PSEN1* and *APP*; compared to non-carriers the adjusted geometric mean was 31% higher (95% CI: 16%, 49%; *P *<* *0.001) in *PSEN1* and 61% higher (95% CI: 44%, 80%; *P *<* *0.001) in *APP* (Fig. 1D). There were also inter-mutation differences in Aβ42:40: The geometric mean was 22% higher (95% CI: 8%, 38%; *P *=* *0.001) in *APP* compared to *PSEN1* carriers.

The geometric mean of Aβ38:40 was higher in *APP* carriers compared to both *PSEN1* carriers (101% higher, 95% CI: 72%, 135%; *P *<* *0.001) and non-carriers (61% higher, 95% CI: 41%, 84%; *P *<* *0.001) (Fig. 1G), while in *PSEN1* Aβ38:40 was reduced compared to non-carriers (geometric mean 20% lower, 95% CI: 10%, 29%, *P *<* *0.001).

For Aβ42:40 ratios, group differences remained significant when separately comparing non-carriers to (i) presymptomatic (18% higher, 95% CI: 3%, 36%, *P *=* *0.02) and symptomatic (47% higher, 95% CI: 23%, 76%, *P *<* *0.001) *PSEN1* carriers; and (ii) presymptomatic (62% higher, 95% CI: 44%, 82%, *P *<* *0.001) and symptomatic (62% higher, 95% CI: 37%, 92%, *P *<* *0.001) *APP* carriers (Fig. 1E and F). Within *PSEN1*, the geometric mean of Aβ42:40 was also 24% higher (95% CI: 2%, 52%; *P *=* *0.03) in symptomatic compared to presymptomatic carriers (Fig. 1E). There were no statistically significant differences between presymptomatic and symptomatic *PSEN1* carriers in Aβ42:38 (*P *=* *0.11; Fig 1B) or Aβ38:40 (*P *=* *0.54; Fig. 1H). Additionally, no significant differences were observed in the Aβ42:40, Aβ42:38 or Aβ38:40 ratios between presymptomatic and symptomatic *APP* carriers (all *P-*values >0.50) (Fig. 1C, F and I).

**Figure 2:**
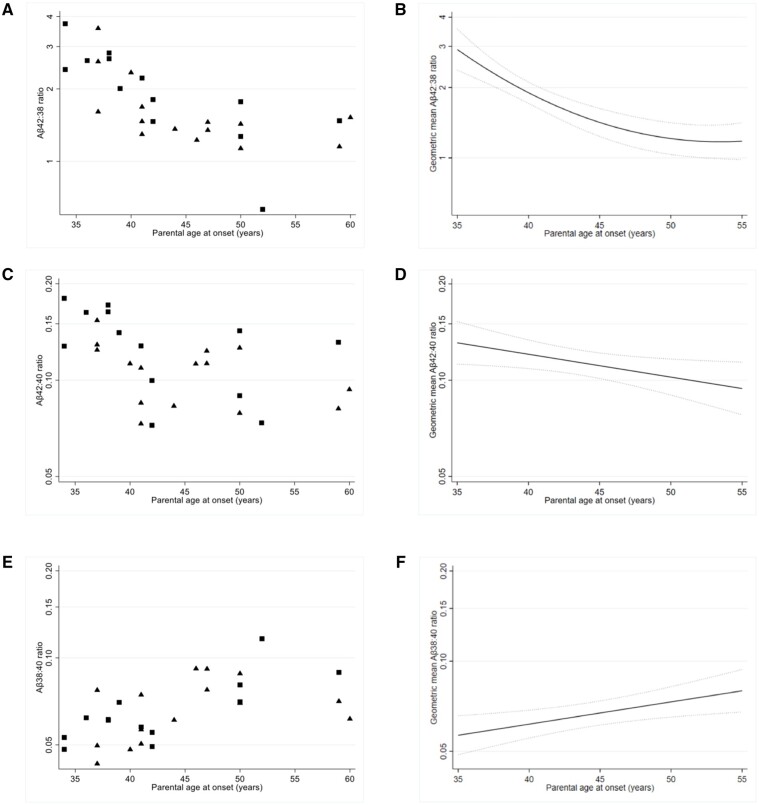
**Plasma amyloid-β ratios against parental AAO in *PSEN1* carriers.** Scatter plots of observed plasma (**A**) Aβ42:38 (**C**) Aβ42:40 and (**E**) Aβ38:40 values against parental age at onset (AAO). Symptomatic mutation carriers are identified by square symbols and presymptomatic mutation carriers by triangle symbols. Modelled geometric mean of plasma (**B**) Aβ42:38 (**D**) Aβ42:40 and (**F**) Aβ38:40 against parental AAO in *PSEN1* carriers; models adjust for EYO, sex and ‘normal ageing’ in non-carriers. The trajectories displayed contain an equal mix of males and females and are adjusted for ‘normal ageing’ relative to age 43 (the average age of mutation carriers). EYO is set at 0, i.e. point of symptom onset, in all three trajectory plots. The *y*-axis scale is logarithmic in all panels.

Using models that adjusted for sex, EYO and ‘normal ageing’, we found significant associations between all three ratios and parental AAO in *PSEN1* carriers (all *P*-values <0.03) (Fig. 2). Higher Aβ42:38 and Aβ42:40 ratios were associated with earlier parental onset, while higher Aβ38:40 was associated with a later disease onset. For Aβ42:38 we included a quadratic term (*P *=* *0.003), which resulted in the estimated rate of change of Aβ42:38 reducing as parental AAO increased; a 1-year increase in parental AAO was associated with a 9.4% decrease (95% CI: 5.3%,13.3%; *P *<* *0.001) in the geometric mean of Aβ42:38 at age 35 compared to a 4.4% decrease (95% CI: 2.9%, 5.9%; *P *<* *0.001) in the same measure at age 45. For both Aβ42:40 and Aβ38:40, the association with parental AAO was estimated to be constant across the age range investigated, a 1-year increase in parental AAO was associated with a 1.6% decrease (95% CI: 0.2%, 3.1%; *P *=* *0.03) in Aβ42:40 and a 1.7% increase (95% CI: 0.4%, 3.0%; *P *=* *0.008) in Aβ38:40. In *APP* carriers, there were no significant associations between Aβ42:40, Aβ42:38 or Aβ38:40 and parental AAO (all *P*-values ≥0.18; Supplementary Fig. 3).

In *PSEN1* and *APP* carriers, models that adjusted for sex, parental AAO and ‘normal ageing’ did not find any significant association between either Aβ42:40, Aβ42:38 or Aβ38:40 and EYO (Supplementary Figs 4 and 5) (*P *≥* *0.06). However, in *APP* carriers there was weak evidence of an association between Aβ42:40 and EYO; a 1-year increase in EYO was associated with a 0.8% decrease (95% CI: 1.6% decrease, 0.0% increase, *P *=* *0.06) in the geometric mean of Aβ42:40.

**Figure 3:**
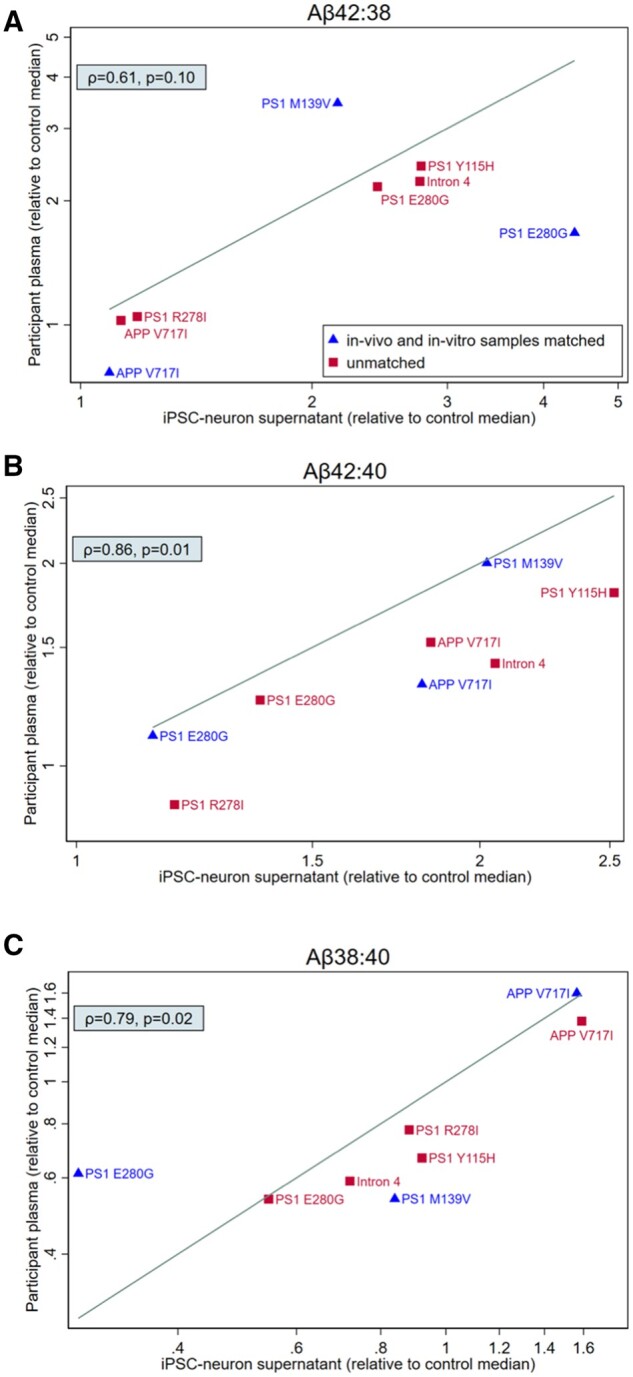
**Comparison of amyloid-β processing *in vivo* and *in vitro.*** Scatterplot comparing amyloid-β ratios profiles in plasma and iPSC-derived neurons for eight mutation carriers. One to one comparison of amyloid-β ratios normalized to the median of controls for each experimental setting (*n* = 27 non-carrier controls for plasma, *n* = 5 iPSC lines from controls who were not members of ADAD families); values >1 indicate higher ratio in a mutation carrier compared to the median of controls, whereas values <1 indicate a lower ratio in a mutation carrier compared to the median of controls. Matched samples (plasma and iPSC samples donated by the same donor) are identified with triangle symbols. Unmatched samples (plasma and iPSC samples donated by different participants who carry the same mutation, and where possible are members of the same family) are identified by square symbols. The *y*-axis scale is logarithmic in all panes. Spearman’s ρ and the associated *P*-value are shown for each scatter plot. The line displayed on each scatterplot represents line of perfect agreement i.e. *x* = *y*.

Amyloid-β ratios in plasma and iPSC-conditioned media were highly associated for both Aβ42:40 (ρ* *=* *0.86, *P *=* *0.01) and Aβ38:40 (ρ* *=* *0.79, *P *=* *0.02), somewhat less so for Aβ42:38 (ρ* *=* *0.61, *P *=* *0.10) (Fig. 3). While we did not observe perfect agreement in the Aβ42:38 ratio between plasma and iPSC lines (shown by a solid line in Fig. 3), the direction of change in this ratio, i.e. either increased or decreased when compared to controls, was largely consistent across media.

## Discussion

In this study, we found increases in plasma Aβ42:40 in both *APP* and *PSEN1* carriers compared to non-carriers and marked differences in amyloid-β ratios between genotypes: Aβ42:38 was higher in *PSEN1* versus *APP*, and Aβ38:40 was higher in *APP* versus *PSEN1*. Importantly, more aggressive *PSEN1* mutations (those with earlier ages of onset) had higher Aβ42:40 and Aβ42:38 ratios—*in* *vivo* evidence of the pathogenicity of these peptide ratios.

These results offer insights into the pathobiology of ADAD and differential effects of *APP*/*PSEN1* genotype. Increased Aβ42:38 in *PSEN1* may be attributed to reduced conversion of Aβ42 (substrate) to 38 (product) relative to non-carriers; in contrast, *APP* carriers showed near identical Aβ42:38 ratios compared to non-carriers. Strikingly, increases in Aβ42 relative to shorter amyloid-β moieties (≤40) were associated with earlier disease onset in *PSEN1.* Importantly, there were no associations between amyloid-β ratios and EYO in *PSEN1* carriers, suggesting these ratios represent molecular drivers of disease as opposed to being markers of disease stage. Our *in* *vivo* results recapitulate cell-based findings of reduced efficiency of γ-secretase processivity in *PSEN1*^6^^,^^10^^,^^11^; inefficiency attributed to impaired enzyme–substrate stability causing premature release of longer amyloid-β peptides.^10^

Parental AAO is an indicator of disease severity, with a younger AAO implying a more deleterious mutation. In *PSEN1*, Aβ42:38 (a read-out of the efficiency of the fourth γ-secretase cleavage) showed a deceleration in the rate of change as parental AAO increases. This further supports the central pathogenic role of γ-secretase processivity in ADAD, especially in younger onset, aggressive forms of *PSEN1*.

In *APP*, production of Aβ38 relative to Aβ40 was increased. This is consistent with a shift in the site of endopeptidase cleavage causing increased generation of Aβ48; the precursor substrate in the Aβ38 production line. Our study included *APP* mutations located near the γ-secretase cleavage site. Previous cell-based work involving mutations around this site also demonstrated increased trafficking along the Aβ48 pathway.^5^^,^^6^^,^^1^,^1^ In contrast, *APP* duplications or mutations near the β-secretase site are associated with non-differential increases in amyloid-β production.^12^

Changes in Aβ38:40 were also seen in *PSEN1* carriers; levels were reduced compared to both *APP* carriers and non-carriers. Declines in Aβ38:40 may reflect mutation effects on endopeptidase cleavage and/or γ-secretase processivity; changes in both processes have been described in *in* *vitro* studies of *PSEN1*.^6^^,^^13^ Premature release of longer (>Aβ43) peptides may contribute to falls in Aβ38:40; both increasing amyloid-β length and pathogenic *PSEN1* mutations are associated with destabilization of the enzyme–substrate complex.^10^ It will be important for future research to investigate the exact molecular drivers of declines in Aβ38:40 in *PSEN1*, especially as lower levels were associated with earlier disease onset.

We also saw inter-stage differences in APP processing; Aβ42:40 was higher in symptomatic compared to presymptomatic *PSEN1* carriers. The reason for this is unclear and should be treated cautiously given the small group sizes and the absence of inter-stage differences in Aβ42:40 among *APP* carriers. However, post-symptomatic increases in plasma Aβ42 have been reported in Down syndrome.^14^ It is possible that downstream pathogenic consequences of ADAD, such as cerebral amyloid angiopathy, may interact with, and modify, plasma levels. Additionally, as amyloid-β is produced peripherally in organs, muscle and platelets, systemic factors may contribute to inter-stage differences.^15^

Our results support the hypothesis that ADAD mutations increase *in* *vivo* production of longer amyloid-β peptides (Aβ ≥ 42) relative to Aβ40. This is consistent with cell- and blood-based studies in ADAD.^1^,^1^^,^^16^ Additionally, we showed plasma amyloid-β profiles were recapitulated in iPSC-media with consistent profiles for the same mutation. There is some evidence that Aβ42:40 ratios also increase in the CSF of mutation carriers far from onset; however, CSF levels then fall significantly during the two decades before symptom onset^17^; reductions are attributed to ‘trapping’ of longer peptides within cerebral plaques.^18^ In sporadic Alzheimer’s disease CSF, as well as plasma, Aβ42:40 levels also fall as cerebral amyloid plaques start to accumulate, with ratio levels remaining low thereafter.^19^ In contrast, we show that plasma Aβ42:40 in both *APP* and *PSEN1* carriers was raised and did not fall below non-carriers’ levels, either before or after symptom onset. Taken together, these findings suggest that plasma amyloid-β ratios in ADAD are less susceptible to the effects of sequestration.

Study limitations include the small sample size, due to the rarity of ADAD; however, we included a reasonably wide array of mutations. Second, ages at onset were estimated from parental AAO, while this offers a reasonable estimate there is variability within families and imprecision in determining AAO in a preceding, often deceased, generation.^20^ Finally, future studies should measure amyloid-β moieties longer than Aβ42, and also investigate interactions between central and peripheral amyloid-β production (we lacked paired CSF).

In conclusion, we demonstrate the impact of pathogenic ADAD mutation on APP processing *in* *vivo*. We show marked inter-mutation difference in Aβ profiles, with relative increases in longer peptides being associated with earlier disease onset. Our findings indicate that plasma amyloid-β ratios in ADAD may be useful biomarkers of APP processing. This is especially important as we enter an era of gene silencing therapies and personalized medicine, where direct read-outs of gene function will be particularly valuable.

## Funding 

A.O. is supported by an Alzheimer’s Society clinical research training fellowship (AS-CTF-18–001), and acknowledges previous support from the Rosetrees Trust. C.A. is supported by a fellowship from the Alzheimer’s Society (AS-JF-18–008) and SW is supported by an Alzheimer’s Research UK Senior Research Fellowship (ARUK-SRF2016B-2). I.J.S. is supported by the UK Dementia Research Institute, which receives its funding from DRI Ltd, funded by the UK Medical Research Council, Alzheimer’s Society and Alzheimer’s Research UK. P.S.J.W. is supported by an MRC Clinical Research Training Fellowship. N.S.R. is supported by a University of London Chadburn Academic Clinical Lectureship. H.Z. is a Wallenberg Scholar supported by grants from the Swedish Research Council (#2018–02532), the European Research Council (#681712), Swedish State Support for Clinical Research (#ALFGBG-720931) and the UK Dementia Research Institute at UCL. K.B. is supported by the Swedish Research Council (#2017–00915), the Alzheimer Drug Discovery Foundation (ADDF), USA (#RDAPB-201809–2016615), the Swedish Alzheimer Foundation (#AF-742881), Hjärnfonden, Sweden (#FO2017-0243) and European Union Joint Program for Neurodegenerative Disorders (JPND2019-466–236). C.F., J.M.N. and T.P.’s academic collaboration with the Dementia Research Centre, UCL is supported by a grant to the DRC from Alzheimer’s Research UK. N.C.F. acknowledges support from Alzheimer's Research UK, the UK Dementia Research Institute and the NIHR UCLH Biomedical Research Centre. This work was supported by the NIHR UCLH/UCL Biomedical Research Centre, the Rosetrees Trust, the MRC Dementia Platform UK and the UK Dementia Research Institute at UCL, which receives its funding from UK DRI Ltd, funded by the UK Medical Research Council, Alzheimer's Society and Alzheimer's Research UK, and the Swedish state under the agreement between the Swedish government and the County Councils, the ALF-agreement (#ALFGBG-715986). N.C.F. had full access to all the data in the study and takes responsibility for the integrity of the data and the accuracy of the data analysis.

## Competing interests

K.B. has served as a consultant at advisory boards or at data monitoring committees for Abcam, Axon, Biogen, JOMDD/Shimadzu, Julius Clinical, Lilly, MagQu, Novartis, Roche Diagnostics and Siemens Healthineers, and is a co-founder of Brain Biomarker Solutions in Gothenburg AB (BBS), which is a part of the GU Ventures Incubator Program. H.Z. has served at scientific advisory boards for Denali, Roche Diagnostics, Wave, Samumed and CogRx, has given lectures in symposia sponsored by Fujirebio, Alzecure and Biogen, and is a co-founder of Brain Biomarker Solutions in Gothenburg AB, a GU Ventures-based platform company at the University of Gothenburg. NCF reports consultancy for Roche, Biogen and Ionis, and serving on a Data Safety Monitoring Board for Biogen. H.R. has undertaken consultancy for Roche.

## Supplementary material

Supplementary material is available at *Brain* online.

## Supplementary Material

awab166_Supplementary_Data

## Abbreviations

AAO: age at onset
Aβ: amyloid-β
ADAD: autosomal dominant Alzheimer’s disease
EYO: estimated years to symptom onset
iPSC: induced pluripotent stem cell
