# Supplementary material for: Plasma amyloid-β ratios in autosomal dominant Alzheimer’s disease: the influence of genotype
Source: Brain. 2021 Apr 23;144(10):2964–70. doi: 10.1093/brain/awab166 (PMC8634092; doi:10.1093/brain/awab166)
Supplement: awab166_Supplementary_Data [file awab166_supplementary_data.zip › awab166-suppl_data/brain-2020-02233-File006.pdf]

	iPSC 42:38	iPSC 42:40	iPSC 38:40	Plasma 42:38	Plasma 42:40	Plasma 38:40
Controls (median (IQR))	0.425 (0.418,0.429) (n=5)	0.110 (0.108,0.111) (n=5)	0.268 (0.258,0.270) (n=5)	1.083 (0.989, 1.154) (n=27)	0.090 (0.800, 0.100) (n=27)	0.085 (0.077, 0.089) (n=27)
APP V717I[a,b]	0.464	0.199	0.419	0.827	0.119	0.144
APP V717I[b]	0.480	0.203	0.426	1.107	0.137	0.124
PSEN1 Y115H[b]	1.180	0.277	0.247	2.624	0.163	0.060
PSEN1 M139V[a,b]	0.918	0.223	0.225	3.733	0.180	0.048
PSEN1 Intron 4[b]	1.175	0.226	0.193	2.407	0.128	0.053
PSEN1 R278I[b]	0.504	0.130	0.236	1.131	0.079	0.070
PSEN1 E280G[a]	1.867	0.126	0.076	1.806	0.100	0.055
PSEN1 E280G	1.034	0.151	0.146	2.338	0.113	0.048

Supplementary Table 1: Measured values of Aβ42:38, Aβ42:40 and Aβ38:40 ratios in paired iPSC lines and plasma samples. Spearman's rho correlations between plasma and iPSC-neuron Aβ ratios are reported in Figure 3 and within the results text of the manuscript.

[a] Matched samples: plasma and iPSC samples donated by the same donor

[b] iPSC data previously published; see (Arber *et al.*, 2019).
