# Supplementary material for: Plasma amyloid-β ratios in autosomal dominant Alzheimer’s disease: the influence of genotype
Source: Brain. 2021 Apr 23;144(10):2964–70. doi: 10.1093/brain/awab166 (PMC8634092; doi:10.1093/brain/awab166)
Supplement: awab166_Supplementary_Data [file awab166_supplementary_data.zip › awab166-suppl_data/brain-2020-02233-File007.pdf]

Appendix 1:
Measurement of plasma Aβ levels

Calibrators were prepared using recombinant Aβ1-38, Aβ1-40 and Aβ1-42 (rPeptide) added to 8% bovine serum albumin in phosphate-buffered saline. Recombinant 15N uniformly labelled Aβ1-38, Aβ1-40 and Aβ1-42 (rPeptide) were used as internal standards (IS), added to samples and calibrators prior to sample preparation. Aβ peptides were extracted from 250 µL human plasma using immunoprecipitation with anti-β-Amyloid 17-24 (4G8) and anti-β-Amyloid 1-16 antibodies (6E10, both BioLegend®) coupled to Dynabeads™ M-280 Sheep Anti-Mouse IgG magnetic beads (Thermo Fisher Scientific). Immunoprecipitation was performed using a KingFisher™ Flex Purification System (Thermo Fisher Scientific). Analysis of processed samples was performed using liquid chromatography-tandem mass spectrometry (LC-MS/MS) on a Dionex Ultimate LC-system and a Thermo Scientific Q Exactive quadrupole-Orbitrap hybrid mass spectrometer. Chromatographic separation was achieved using basic mobile phases and a reversed-phase monolith column at a flow rate of 0.3 mL/min. The mass spectrometer operated in parallel reaction monitoring (PRM) mode was set to isolate the 4+ charge state precursors of the Aβ peptides. Product ions (14-15 depending on peptide) specific for each precursor was selected and summed to calculate the chromatographic areas for each peptide and its corresponding IS. The area ratio of the analyte to the internal standard in unknown samples and calibrators was used for quantification.
