# Supplementary material for: Plasma amyloid-β ratios in autosomal dominant Alzheimer’s disease: the influence of genotype
Source: Brain. 2021 Apr 23;144(10):2964–70. doi: 10.1093/brain/awab166 (PMC8634092; doi:10.1093/brain/awab166)
Supplement: awab166_Supplementary_Data [file awab166_supplementary_data.zip › awab166-suppl_data/brain-2020-02233-File008.pdf]

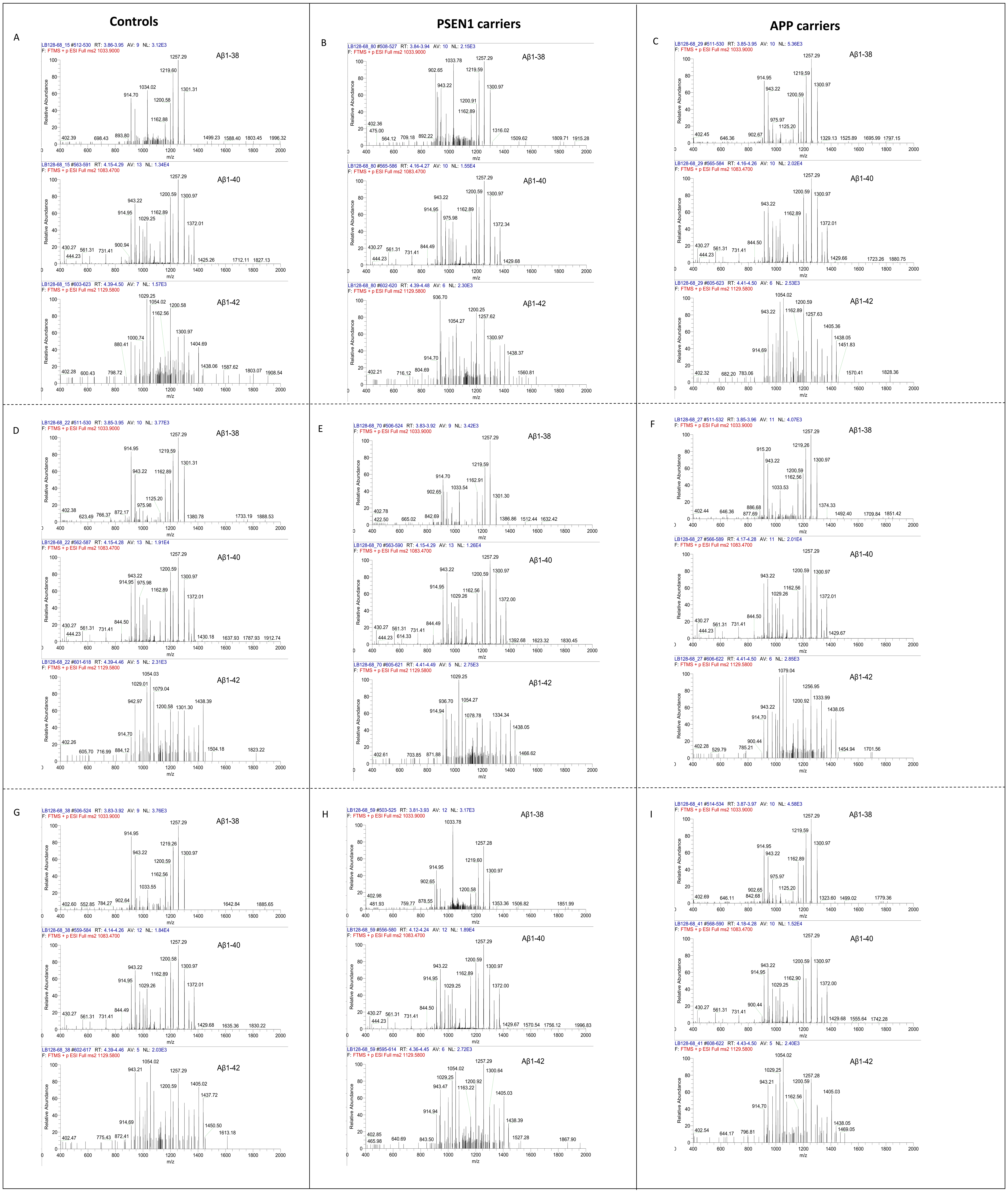

Supplementary figure 1: Representative mass spectrometry (MS) spectra for Aβ38, Aβ40 and Aβ42.
Representative MS profiles for 3 non-carrier controls **(1A, 1D, 1G)**, 3 *PSEN1* carriers **(1B, 1E, 1H)** and *APP* carriers **(1C, 1F, 1I)**. Panes **1B** and **1C** display profiles of presymptomatic mutation carriers, while panes **1E, 1F,1H, 1I** display profiles of symptomatic mutation carriers.
