# Supplementary material for: Plasma amyloid-β ratios in autosomal dominant Alzheimer’s disease: the influence of genotype
Source: Brain. 2021 Apr 23;144(10):2964–70. doi: 10.1093/brain/awab166 (PMC8634092; doi:10.1093/brain/awab166)
Supplement: awab166_Supplementary_Data [file awab166_supplementary_data.zip › awab166-suppl_data/brain-2020-02233-File010.pdf]

Gene	Mutation	Number of individuals
APP	p.Thr719Asn	1 AR
	p.Val717Gly	2 S
	p.Val717Ile	1 S, 7 AR
	p.Val717Leu	2 S, 2 AR
PS1	Intron 4	2 S, 5 AR
	p.Ala79Val	1 S
	p.Tyr115His	1 S, 1 AR
	p.Glu120Lys	1 AR
	p.Ser132Ala	2 AR
	p.Met139Val	1 S, 1 AR
	p.Val142Ile	1 S
	p.Met146Ile	2 AR
	p.Glu184Asp	2 S, 4 AR
	p.Ile202Phe	4 AR
	p.Gly206Ala	1 S
	p.His214Tyr	3 AR
	p.Ala246Glu	2 AR
	p.Pro264Leu	2 AR
	p.Pro267Ser	1 S
	p.Arg269His	1 AR
	p.Arg278Ile	3 AR,
	p.Glu280Gly	2 S, 6 AR,
	ΔE9*	1 S

Supplementary Table 2: The number of individuals from families with each mutation is given, divided in to symptomatic (S) or asymptomatic but at risk (AR). Details relating to how many at risk participants for each mutation were mutation carriers is not given to ensure it is not possible the mutation status of any at risk individual to be revealed/deduced. ** The exon 9 deletion (NM_000021.3:c.869-1G>T; p.Ser290Cys;Thr291_Ser319del) commonly referred to as ΔE9. *APP* mutations investigated lie near the γ-secretase cleavage site.
