# Supplementary material for: Plasma amyloid-β ratios in autosomal dominant Alzheimer’s disease: the influence of genotype
Source: Brain. 2021 Apr 23;144(10):2964–70. doi: 10.1093/brain/awab166 (PMC8634092; doi:10.1093/brain/awab166)
Supplement: awab166_Supplementary_Data [file awab166_supplementary_data.zip › awab166-suppl_data/brain-2020-02233-File011.pdf]

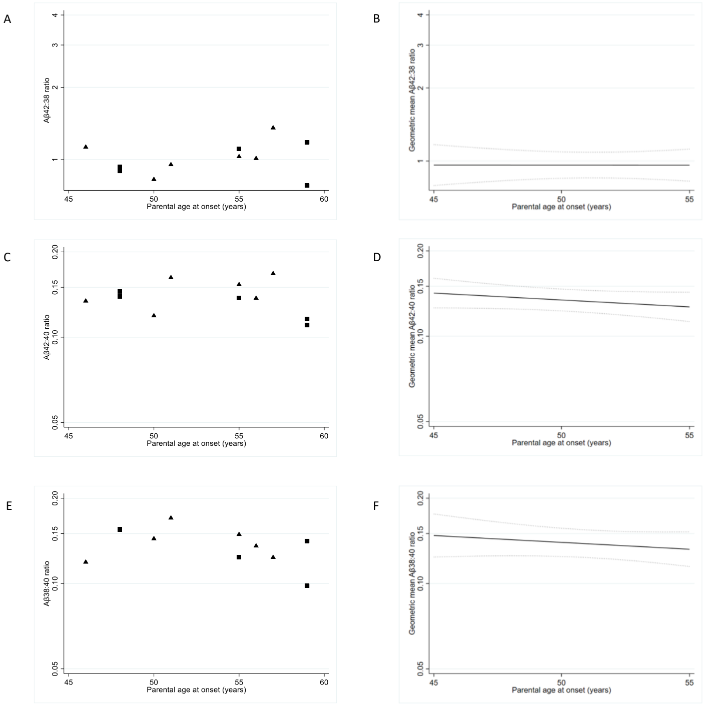

Supplementary figure 3: Plasma Aβ ratios against parental AAO in *APP* carriers.
Scatter plots of observed plasma **(A)** Aβ42:38 **(C)** Aβ42:40 and **(E)** Aβ38:40 values against parental AAO. All scatter plots show values for *APP* carriers only. Symptomatic mutation carriers are identified by square symbols and presymptomatic mutation carriers by triangle symbols. Modelled geometric mean of plasma **(B)** Aβ42:38 **(D)** Aβ42:40 and **(F)** Aβ38:40 against parental AAO in *APP* carriers. The trajectories displayed contain an equal mix of males/females and are adjusted to 'normal ageing' in non-carriers relative to age 43 (average age of mutation carriers). EYO is set at 0, i.e. point of symptom onset, in all three trajectory plots. Models, which adjusted for EYO, sex and 'normal ageing', did not show evidence of any significant associations between either Aβ42:38, Aβ42:40 or Aβ38:40 and parental AAO: for Aβ42:38 a one-year increase in parental AAO was associated with an estimated 0.0% decrease (95% CI: 2.4% decrease, 2.4% increase; p=1.00); for Aβ42:40 an estimated 1.1% decrease (95% CI: 2.7% decrease, 0.50% increase; p=0.18); for Aβ38:40 an estimated 1.1% decrease (95% CI: 3.3% decrease, 1.1% increase; p=0.32). The y-axis scale is logarithmic in all panes.
