# Supplementary material for: Plasma amyloid-β ratios in autosomal dominant Alzheimer’s disease: the influence of genotype
Source: Brain. 2021 Apr 23;144(10):2964–70. doi: 10.1093/brain/awab166 (PMC8634092; doi:10.1093/brain/awab166)
Supplement: awab166_Supplementary_Data [file awab166_supplementary_data.zip › awab166-suppl_data/brain-2020-02233-File012.pdf]

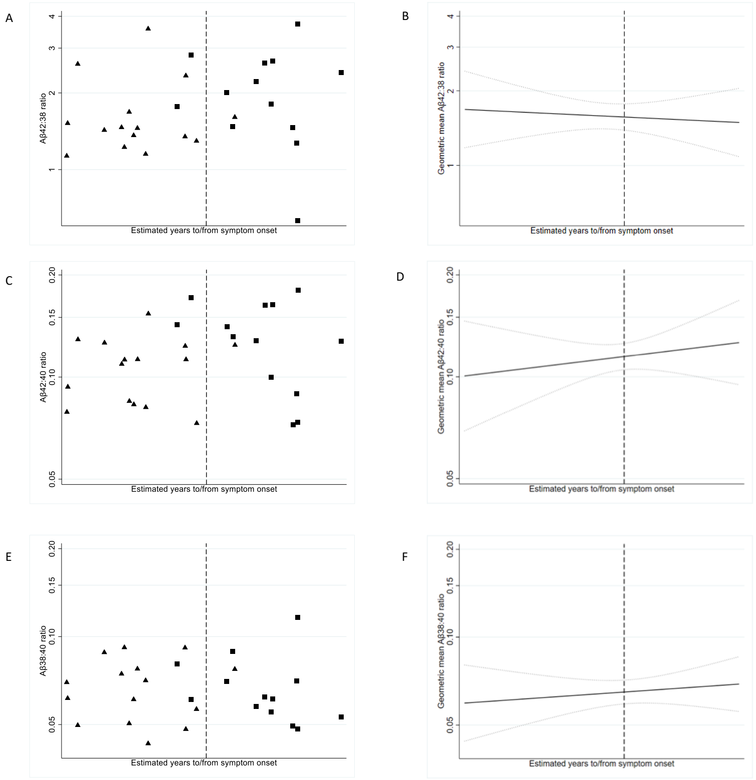

Supplementary figure 4: Plasma Aβ ratios against estimated years to/from symptom onset (EYO) in *PSEN1* carriers.

Scatter plots of observed plasma **(A)** Aβ42:38 **(C)** Aβ42:40 and **(E)** Aβ38:40 values against EYO. All scatter plots show values for *PSEN1* carriers only. Symptomatic mutation carriers are identified by square symbols and presymptomatic mutation carriers by triangle symbols.

Modelled geometric mean of plasma **(B)** Aβ42:38 **(D)** Aβ42:40 and **(F)** Aβ38:40 against EYO in *PSEN1* carriers. The trajectories displayed contain an equal mix of males/females and are adjusted to 'normal ageing' in non-carriers relative to age 43 (average age of mutation carriers). Parental AAO is set at 43 in all three trajectory plots. Models, which adjusted for parental AAO, sex and 'normal ageing', did not show evidence of any significant associations between either Aβ42:38, Aβ42:40 or Aβ38:40 and EYO: for Aβ42:38 a one-year increase in EYO was associated with an estimated 0.3% decrease (95% CI: 1.7% decrease, 1.2% increase; p=0.71); for Aβ42:40 an estimated 0.5% increase (95% CI: 0.9% decrease, 2.0% increase; p=0.48); for Aβ38:40 an estimated 0.3% increase (95% CI: 0.8% decrease, 1.5% increase; p=0.54). To maintain blinding of mutation status, the values of the x-axis for all EYO plots have been removed. The y-axis scale is logarithmic in all panes.
